# Improving the Selectivity of ZIF-8/Polysulfone-Mixed Matrix Membranes by Polydopamine Modification for H_2_/CO_2_ Separation

**DOI:** 10.3389/fchem.2020.00528

**Published:** 2020-07-10

**Authors:** Xueyi Mei, Sheng Yang, Peng Lu, Yexin Zhang, Jian Zhang

**Affiliations:** ^1^Key Laboratory of Bio-based Polymeric Materials Technology and Application of Zhejiang Province, Ningbo Institute of Materials Technology and Engineering, Chinese Academy of Sciences (CAS), Ningbo, China; ^2^University of Chinese Academy of Sciences, Beijing, China; ^3^School of Materials Science and Chemical Engineering, Ningbo University, Ningbo, China

**Keywords:** polysulfone, mixed matrix membranes, ZIF-8 nanoparticles, polydopamine, H_2_/CO_2_separation

## Abstract

Gas separation membranes are essential for the capture, storage, and utilization (CSU) of CO_2_, especially for H_2_/CO_2_separation. However, both glassy and rubbery polymer membranes lead a relatively poor selectivity for H_2_/CO_2_ separation because the differences in kinetic diameters of these gases are small. The present study establishing the mixed matrix membranes (MMMs) consist of a nano-sized zeolitic imidazolate frameworks (ZIF-8) blended with the polysulfone (PSf) asymmetric membranes. The gas transport properties (H_2_, CO_2_, N_2_, and CH_4_) of MMMs with a ZIF-8 loading up to 10 wt% were tested and showing significant improvement on permeance of the light gases (e.g., H_2_ and CO_2_). Moreover, the depositional polydopamine (PDA) layer further enhanced the ideal H_2_/CO_2_ selectivity, and the PDA-modified MMMs approach the Robeson upper bound of H_2_/CO_2_ separation membranes. Hence, the PDA post-modification strategy can effectively repair the defects of MMMs and improved the H_2_/CO_2_selectivity.

## Introduction

The continuous rise of atmosphere carbon dioxide (CO_2_) concentration caused by excessive anthropological combustion of fossil fuels leads to global warming and extreme climate events (Gao et al., 2017). In this situation, capture, storage, and utilization (CSU) of CO_2_ from other sources have been a worldwide attention (Zhao et al., 2016; Zheng et al., 2016). At present, there are three technically plausible strategies for CO_2_ CSU: post-combustion CO_2_ capture (mainly for CO_2_/N_2_ separation), oxy-combustion (mainly for O_2_/N_2_ separation), and pre-combustion CO_2_ capture (mainly for H_2_/CO_2_ separation; Ramasubramanian et al., 2013; Yan et al., 2015). Among them, pre-combustion CO_2_ capture is a promising technology, which can reduce the CO_2_ emission and mitigate energy crisis (Wang et al., 2012; Liao et al., 2015). Since the H_2_/CO_2_ syngas from water–gas shift reaction can provide H_2_ as a preferred fuel or chemical feedstock, it is very important for the separation technologies for H_2_/CO_2_.

Membrane separation technology has obtained a great deal of attention for H_2_/CO_2_ separation due to the fewer environmental impacts and lower energy costs, compared to conventional industrial methods (e.g., adsorption or cryogenic distillation; Rabiee et al., 2014; Liu et al., 2016; Wang et al., 2017; Ibrahim and Lin, 2018). Besides, membrane separation processes can be employed for the capture of CO_2_, while H_2_ is subjected to combustion, which is due to a very high permeation rate of H2 relative to most other gases (Fu et al., 2016). However, polymer membrane performance has been limited by a trade-off between gas permeation and selectivity, known as “Robeson upper bound” (Chua et al., 2011; Zhang et al., 2012; Sánchez Laínez et al., 2018a). Both glassy and rubbery polymer membranes show a relatively poor selectivity for separation of H_2_/CO_2_ because the differences in kinetic diameters of these gases are small (2.89 Å for H_2_ and 3.3 Å for CO_2_; Hosseini et al., 2010; Kim et al., 2018). The inorganic membranes generally have uniform pore size and excellent resistance to high temperature and pressure, e.g., silica membranes (Song et al., 2016a,b), which can achieved both high permeability and selectivity (Xiang et al., 2017). However, the expensive price limited the large-scale gas application of inorganic membranes. Recent gas separation membranes have been focused on the mixed matrix membranes (MMMs), which compensated the limitations of polymeric and inorganic membranes, while offering an ease in processability and moderate processing cost (Nordin et al., 2015).

Zeolitic imidazolate frameworks (ZIFs) are promising materials for gas separation membrane fabrication, for example, zeolitic imidazolate framework (ZIF-8) as one of the most investigated MOFs with the sod topology and the smaller window of 3.4 Å, which is close to the kinetic diameter of H_2_ (2.89 Å; Sánchez Laínez et al., 2018b). A highly oriented ZIF-8 membrane on a porous α-alumina support was reported by Bux et al. (2011). The results showed that the H_2_ permeance of ZIF-8 membrane was ~4,032 Barrer, while the H_2_/CO_2_ selectivity was only six. Besides, the pure ZIF-8 membranes are difficult to be reproduced on a large-scale and are too brittle to withstand high operating pressures (Gascon et al., 2012). Recently, the number of studies focused on the utilization of ZIF-8 for MMM preparation, which could potentially overcome the H_2_/CO_2_ Robeson upper bound of gas separation membranes (Li et al., 2015). Song et al. (2012) incorporated the ZIF-8 as a nanofiller into a model polymer matrix (Matrimid® 5,218) via a mixing solution, showing enhanced permeability of the MMMs with negligible loss in selectivity. Wijenayake et al. (2013) fabricated a polyimide MMM with 33.3 wt% ZIF-8, and H_2_ permeability of prepared MMM showed an approximate 400% improvement. Nevertheless, excessive ZIF-8 loadings would increase the chances to agglomerate and increase the defective risks of MMMs.

**Figure 1:**
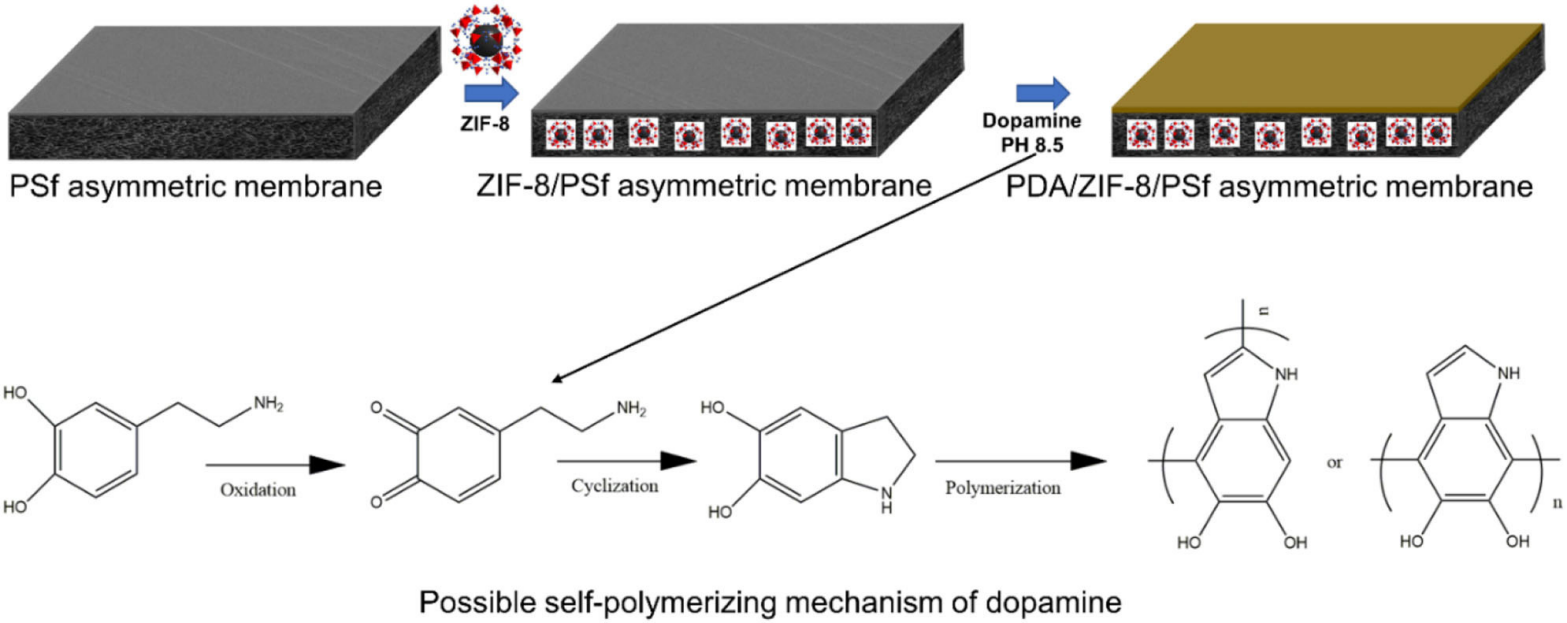
Schematic diagram of preparation of polydopamine (PDA)-modified zeolitic imidazolate frameworks (ZIF-8)/polysulfone (PSf) mixed matrix membranes (MMMs).

Developing defect-free ZIF-8/polymer MMMs is a major challenge because the defect leads to the deterioration of the membrane performance (Dechnik et al., 2017). Recently, surface post-treatment could effectively repair defects of MMMs (Nordin et al., 2014). Polydopamine (PDA), which is prepared through dopamine self-polymerization in weak alkaline solutions with the participation of oxygen, forming a PDA coating adhere onto nearly all kinds of substrates, has drawn much attention. (Lu et al., 2017; Yang et al., 2018). This work aims to develop a new type of asymmetric MMMs via phase inversion and PDA modification (Figure 1), further improving the H_2_/CO_2_ selectivity. By using our designed strategy, the gas permeance of ZIF-8/PSf MMMs was significantly improved in the presence of a certain amount of ZIF-8 nanoparticles. Furthermore, the effect of polydopamine (PDA)-modified MMMs on the gas transport was studied.

## Experiment

### Materials

PSf (Mn: 22,000 Da) and PDA (98.5%) were purchased from Sigma-Aldrich. Zinc nitrate hexahydrate [Zn(NO_3_)_2_•6H_2_O, 98%], 2-methylimidazole (mIm, 99%), N-N-dimethyl-acetamide (DMAC), tetrahydrofuran (THF), methanol, and ethanol (EtOH) were purchased from Sinopharm Chemical Reagent Co., Ltd. All chemicals were used without further purification.

### Synthesis of ZIF-8 Nanoparticles

ZIF-8 nanoparticles were obtained by the solvent method as described in a previous study (Sánchez-Laínez et al., 2016). First, 1.47 g of Zn(NO_3_)_2_•6H_2_O and 3.25 g of mIm were added in 100 ml of MeOH as solutions A and B, respectively. Then, solution A was rapidly poured into solution B under stirring. The mixture solution was reacted for 30 min with continuous stirring at room temperature. Finally, the ZIF-8 nanocrystals were separated from the milky dispersion by centrifugation and then were washed with fresh MeOH three times. The obtained ZIF-8 nanoparticles were directly used for the preparation of MMMs without drying.

### Preparation of PDA-Modified ZIF-8/PSf MMMs

Asymmetric PSf membranes were fabricated by a phase separation method as described in the previous study (Ismail et al., 2003), which consist of 22.0 wt% PSf polmyer, 31.8 wt% DMAC, 31.8 wt% THF, and 14.4 wt% EtOH. For asymmetric PSf membranes, the only difference is the evaporation time (30, 45, 60, and 90 s), and the obtained samples were defined as PSf-30, PSf-45, PSf-60, and PSf-90 membranes. For ZIF-8/PSf MMMs, a certain amount (2.5, 5, 7.5, 10, and 15 wt%) of ZIF-8 was added into the mixture solvents under stirring, followed by 30 min of ultrasonication. Then, the PSf polymer was dissolved in a solution mentioned above and kept stirring for 8 h. Next, the dope solution was casted on a glass plate with a 150-μm casting knife (Elcometer3530). The as-prepared MMMs were immersed into a DI-water bath for 24 h after the 30-s evaporation at room temperature. The MMMs were dried for 24 h in ambient atmosphere and defined as 2.5 wt% ZIF-8/PSf-30, 5 wt% ZIF-8/PSf-30, 7.5 wt% ZIF-8/PSf-30, 10 wt% ZIF-8/PSf-30, and 15 wt% ZIF-8/PSf-30. The PDA coating layer could reduce the defects of MMMs. The as-prepared ZIF-8/PSf MMMs were immersed into a PDA Tris buffer solution (2 mg ml^−1^, 10 mM, and pH = 8.5) with different coating times (0.5, 1, 1.5, 2, 2.5, and 3 h). Then, the PDA-modified MMMs were rinsed with DI-water and dried in a vacuum oven for 12 h at room temperature.

### Characterization

An XRD diffractometer (Bruker D8, Germany) was used to detect the crystal structure of the ZIF-8 at 2θ = 5°-40° with 0.02 step size. The infrared spectral analysis of the ZIF-8 nanoparticles and MMMs were tested by attenuated total reflectance-Fourier transform infrared spectroscopy (ATR-FTIR; Vertex 70; Bruker). The morphology and structure of the samples were observed by scanning electron microscopy (Nova NanoSEM450, USA). The N_2_ adsorption and desorption isotherms of the ZIF-8 nanoparticles were observed by 3Flex physical adsorption instrument (Micromeritics, USA) at 77 K, and the adsorption isotherms of H_2_ and CO_2_ were tested at 273 K. A thermogravimetric analyzer (TGAQ50; TA Instruments-Waters LLC) was used for evaluating the thermal stability of the ZIF-8 nanoparticles and MMMs. All samples were heated from 30 to 800°C with 10°C min^−1^ heating rate under N_2_ with a flow of 50-ml min^−1^.

### Gas Permeation Tests

Single-gas permeability of membranes was measured using a constant-volume variable-pressure method (Zhao et al., 2018). The entire permeation cell was placed in an oven to keep the temperature at 30°C. The permeation cell was kept under vacuum for 12 h to remove other gases. The effective area of membranes is about 0.3 cm^2^, and the gas permeability of each samples has been tested at least three times. The gas permeation (in terms of Barrer, 1 Barrer = 1 × 10^−10^ cm^3^ (STP) cm cm^−2^ s^−1^ cmHg^−1^) was calculated by the following Equation (1):

(1) $$\begin{array}{l} P=\left(\frac{273\times 10^{10}}{760}\right)\left[\frac{Vl}{AT\left(P_{0}\times \frac{76}{14.7}\right)}\right]\left(\frac{dp}{dt}\right) \end{array}$$

where, *P* is the gas permeability in Barrer, *V* is the constant volume container (cm^3^), *l* is the thickness of dense layer of membrane (cm), which are obtained from SEM images. *A* is the membrane surface area (cm^2^), *T* is the temperature (K), *P*_0_ is the upstream (feed) pressure (psia), and *dp/dt* is the change in pressure against time (mmHg/s). The gas ideal selectivity (α_*i*/*j*_) for components *i* and *j* was defined as the ratio of gas permeability of the two components by the following Equation (2):

(2) $$\begin{array}{l} \alpha_{i/j}=\frac{P_{i}}{P_{j}} \end{array}$$

## Results and Discussion

### Characterization of ZIF-8 Nanoparticles

**Figure 2:**
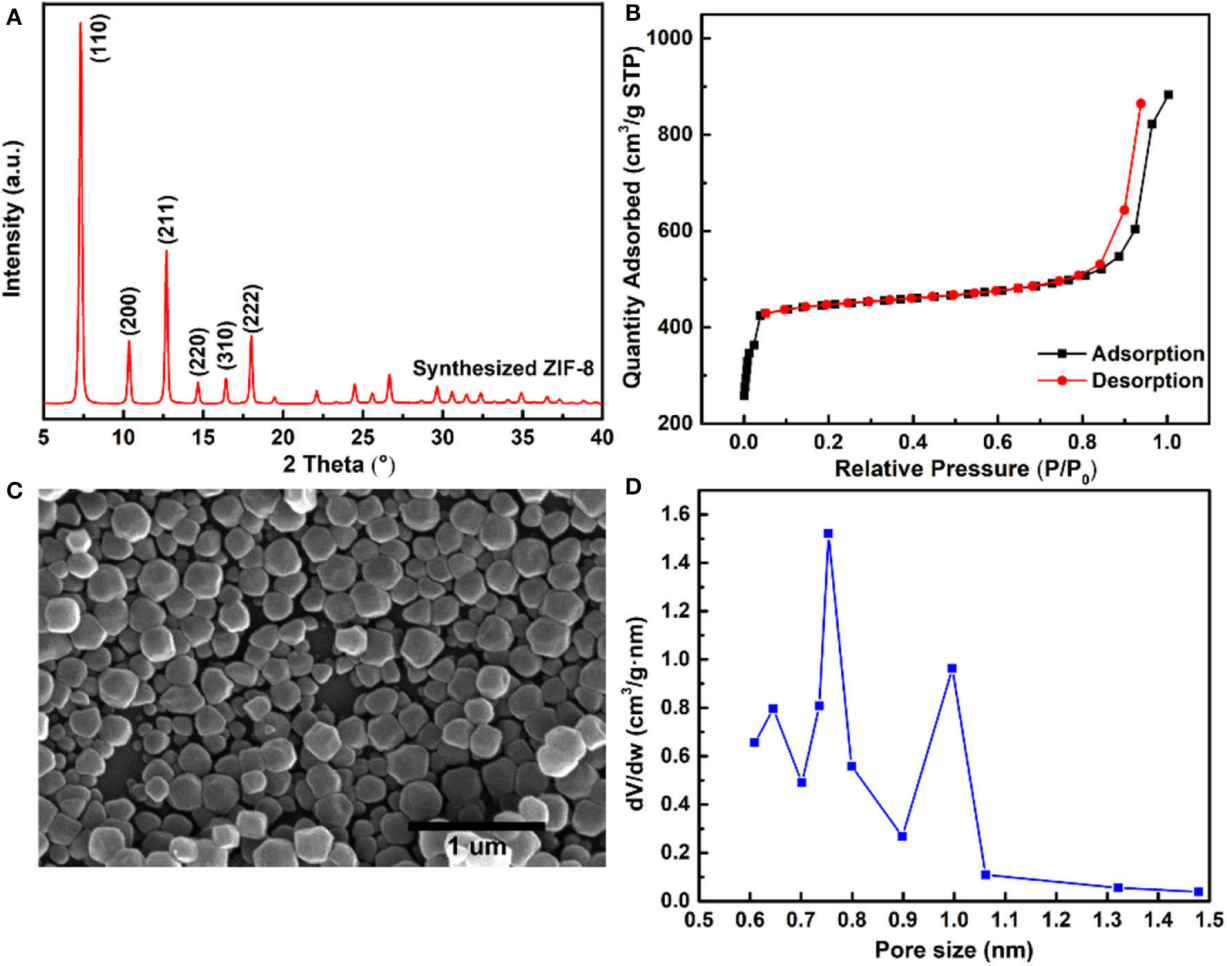
**(A)** XRD pattern of ZIF-8 nanoparticles; **(B)** SEM image of ZIF-8 nanoparticles; **(C)** N_2_ adsorption–desorption isotherms; and **(D)** pore size distribution of the ZIF-8 nanoparticles.

The ZIF-8 nanoparticles were synthesized by the solvent method, and the XRD characteristic peaks of ZIF-8 are shown in Figure 2A, comfirming the typical sodalite (SOD) type structure of ZIF-8 nanoparticles (Park et al., 2006; Cravillon et al., 2009). Figure 2B shows the resembling spherical morphology of ZIF-8 nanoparticles by SEM characterization, and the average size of ZIF-8 nanoparticles was about 150 nm. The microporous structure of ZIF-8 was confirmed by N_2_ adsorption and desorption isotherms, and the ZIF-8 nanoparticles exhibited a typical Type-I isotherm, as shown in Figure 2C. The BET surface area and pore volume of ZIF-8 nanoparticles were 1,371 m^2^ g^−1^ and 0.72 cm^3^ g^−1^, respectively. The pore size distribution (PSD) provided further insight into the pore structure of ZIF-8 nanoparticles (Figure 2D). The 0.64 and 0.75 nm PSD centers of ZIF-8 represented the flexible six-membered ring, which arises from the vibrations of imidazole ligands (Guo et al., 2018). In addition, the largest PSD center of ZIF-8 was about 1.0 nm, which corresponds to the diameter of the ZIF-8 SOD cage.

### Effect of Evaporation for Asymmetric PSf Membranes

**Figure 3:**
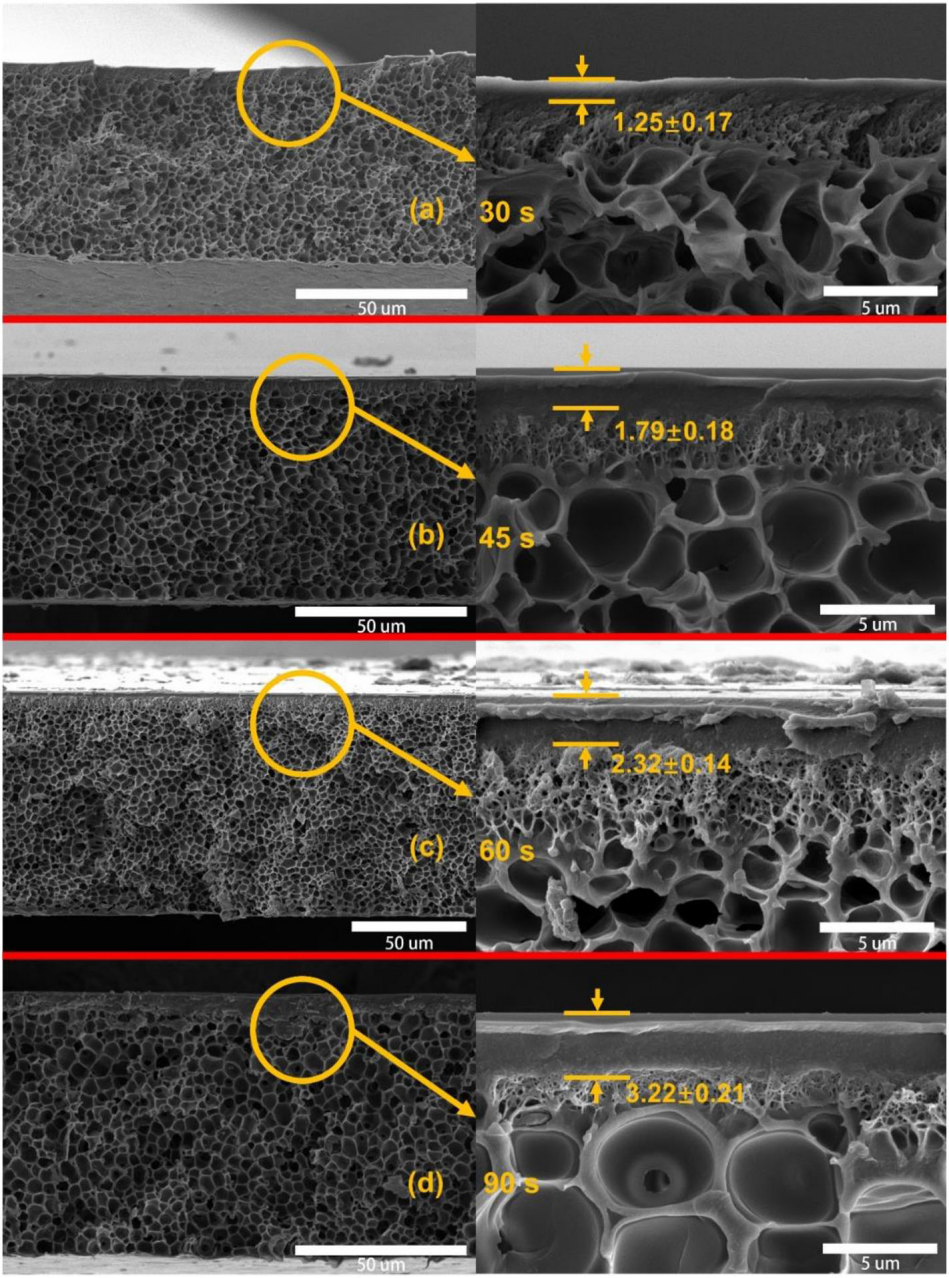
Cross-section SEM images of the asymmetric polysulfone (PSf) membranes with different evaporation times. **(a)** 30 s; **(b)** 45 s; **(c)** 60 s; and **(d)** 90 s.

To optimize the preparation process of asymmetric PSf membranes, the effect of evaporation for gas separation performance of membranes was investigated as shown in Table 1. It can be found that the H_2_ and CO_2_ permeabilities significantly were reduced with the increasing evaporation time. This is due to the evaporation process that induced the skin layer formed of PSf membranes, and the skin layer thickness was improved with increasing evaporation times as shown in Figure 3. The formed skin layer is a resistance barrier between the PSf membrane and the coagulation bath. Presence of this resistance barrier induced the densification of skin layer as the evaporation time (Hołda et al., 2013). Moreover, all asymmetric PSf membranes exhibited lower N_2_ and CH_4_ gas permeabilities than the other gases, owning to their larger kinetic diameters. The cross-section morphology of asymmetric PSf membranes with different evaporation times is shown in Figure 3, which consist of the extremely well-defined dense skin layers supported on a highly open-celled structure. Based on the gas separation test results, the PSf-30 membrane was used for the ZIF-8/PSf MMM preparation.

**Table 1:** Gas permeability and selectivity of asymmetric polysulfone (PSf) membranes with different evaporation time at 4 bar and 30°C.

**Membranes**	**Selectivity**				**Permeability (Barrer)**			
	**H_**2**_/N_**2**_**	**H_**2**_/CO_**2**_**	**CO_**2**_/CH_**4**_**	**CO_**2**_/N_**2**_**	**N_**2**_**	**CH_**4**_**	**CO_**2**_**	**H_**2**_**
PSf-30	47.90	2.18	20.88	21.95	0.78	0.82	17.12	37.36
PSf-45	48.33	2.32	17.86	20.83	0.48	0.56	10.00	23.20
PSf-60	42.46	2.25	18.84	18.84	0.52	0.52	9.80	22.08
PSf-90	36.25	2.40	15.00	15.00	0.48	0.48	7.20	17.40

### Effect of ZIF-8 Loading for ZIF-8/PSf-30 MMMs

**Figure 4:**
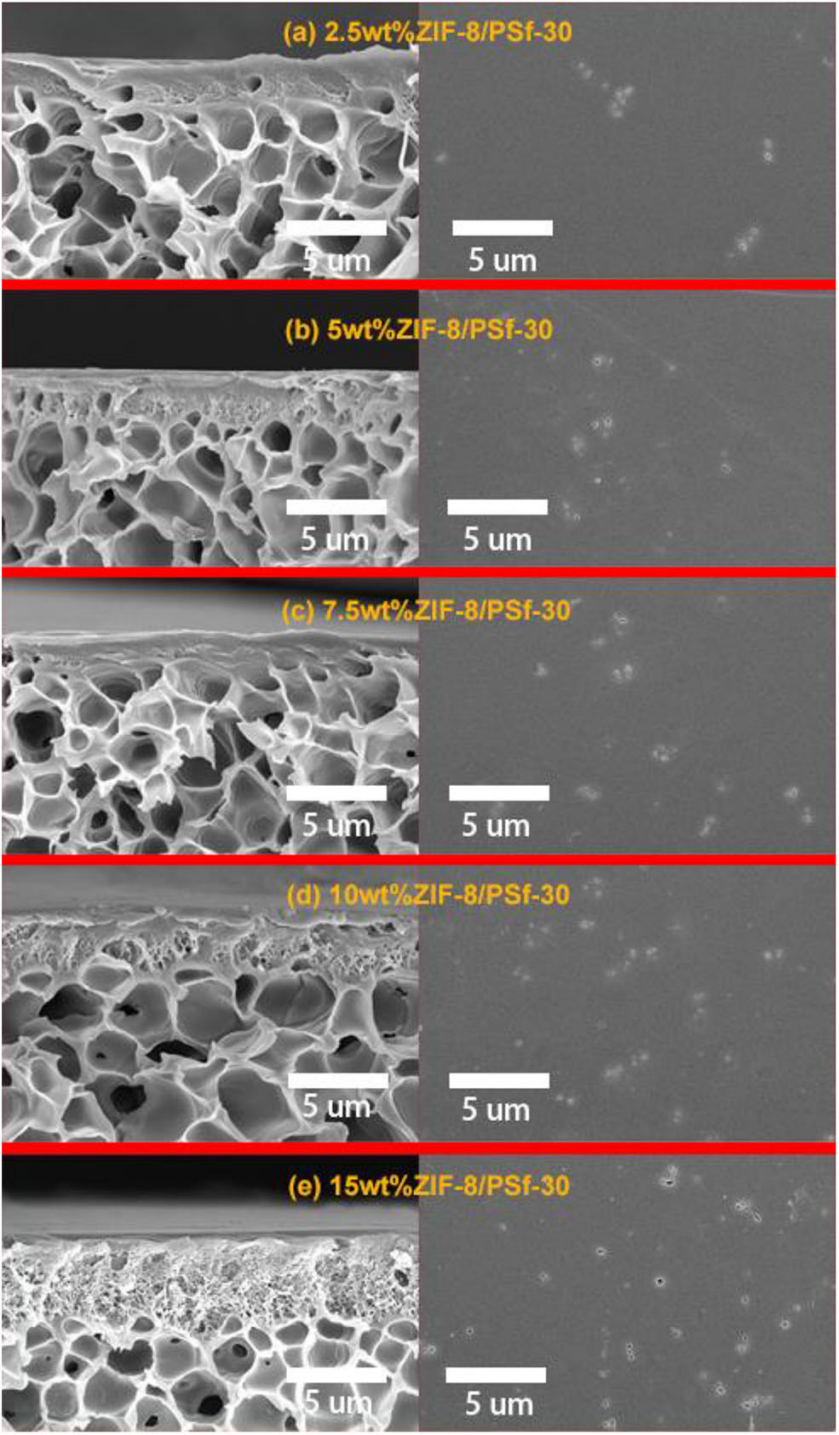
Cross-section and surface of SEM images of ZIF-8/PSf-30 MMMs with different ZIF-8 loadings. **(a)** 2.5 wt%; **(b)** 5 wt%; **(c)** 7.5 wt%; **(d)** 10 wt%; and **(e)** 15 wt%.

Table 2 summarizes the effects of the ZIF-8 gas loading on the gas separation performance of MMMs. Compared with the asymmetric PSf-30 membrane, it was confirmed that permeability of MMMs for the four gases was improved with increasing ZIF-8 loading below 10 wt.%. However, an excessive amount of ZIF-8 can cause the separation performance to decline in the case of the 15 wt% ZIF-8/PSf-30 MMM. Figure 4 shows the cross-section and top surface of the five different MMMs. The surface defect was significantly increased due to the incorporation of ZIF-8 with different loadings. The defects on the surface of MMMs could be attributed to the ZIF-8 agglomeration (Nafisi and Hägg, 2014; Boroglu and Yumru, 2017). Owing to these defects of surfaces, the gas selectivity of MMMs did not change significantly; only the improvement of H_2_/N_2_ selectivity was found. In addition, the thickness of sublayers under the dense skin layers was improved (Figure 4) with the increasing ZIF-8 loading. This morphological change in the MMMs was attributed to the delayed demixing during the phase separation, leading to the dense skin layer transformation to the porous layer (Lu et al., 2016).

**Table 2:** Gas permeation of zeolitic imidazolate frameworks (ZIF-8)/polysulfone (PSf)-30 mixed matrix membranes (MMMs) with different ZIF-8 loading at 4 bar and 30°C.

**ZIF-8 loading (%)^a^**	**Selectivity**				**Permeability (Barrer)**			
	**H_**2**_/N_**2**_**	**H_**2**_/CO_**2**_**	**CO_**2**_/CH_**4**_**	**CO_**2**_/N_**2**_**	**N_**2**_**	**CH_**4**_**	**CO_**2**_**	**H_**2**_**
2.5	45.59	2.07	22.00	22.00	0.88	0.88	19.36	40.12
5	57.91	2.30	24.82	25.08	0.92	0.93	23.08	53.28
7.5	51.75	1.97	26.31	25.33	1.30	1.35	34.20	67.28
10	65.91	2.38	27.72	26.52	1.32	1.38	36.60	87.00
15	41.96	2.28	17.75	18.41	1.08	1.12	19.88	45.32

a *The mass of ZIF-8 based on the mass of PSf polymer*.

### PDA Coating the 10 Wt% ZIF-8/PSf-30 MMMs

**Figure 5:**
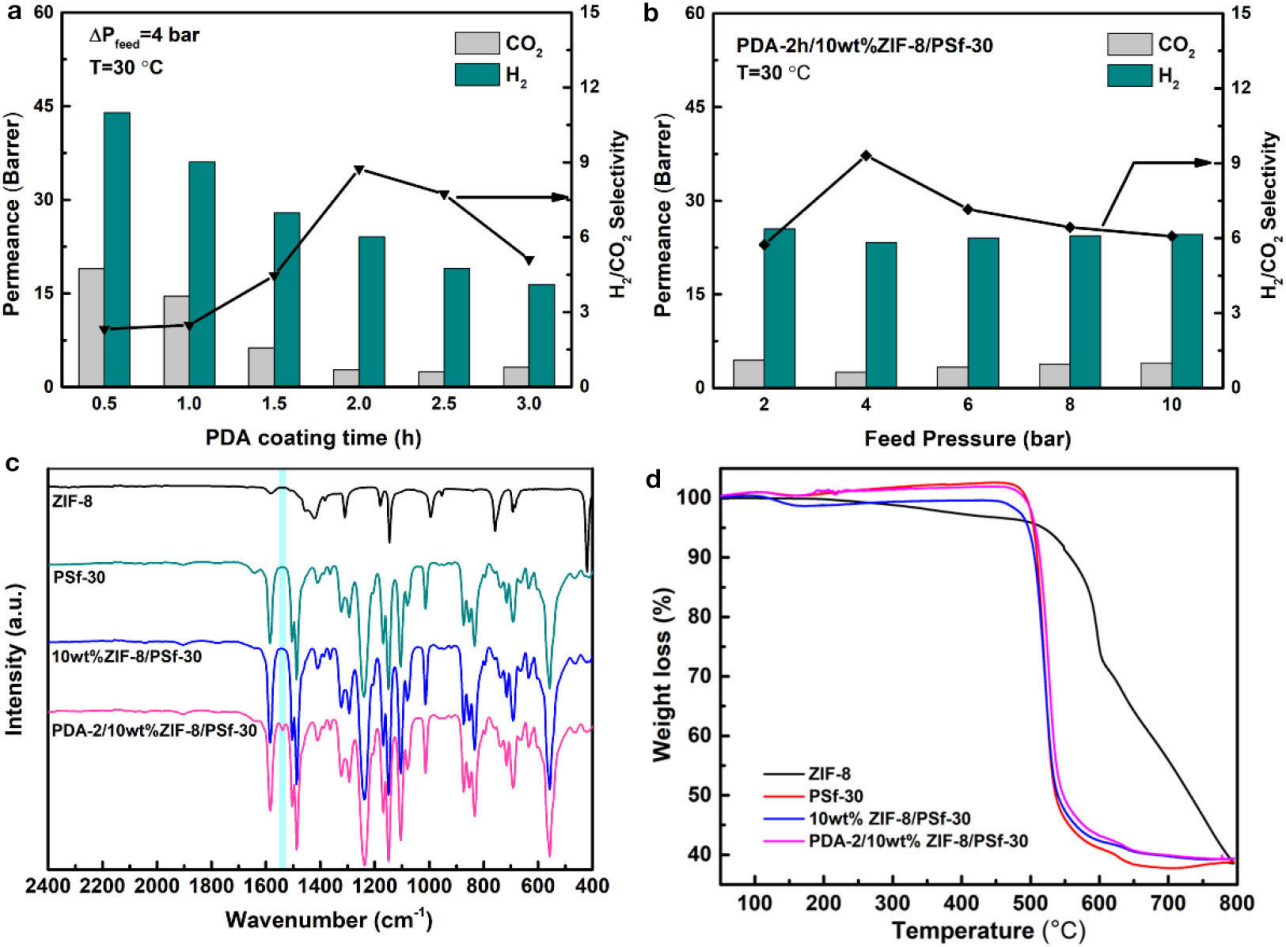
**(a)** Effect of different PDA coating time of the 10 wt% ZIF-8/PSf-30 MMM on H_2_/CO_2_ separation at 30°C under 4 bar; **(b)** Effect of feed pressures on the H_2_/CO_2_ separation of PDA-2/10 wt% ZIF-8/PSf-30 MMM at 30°C; **(c)** attenuated total reflectance-Fourier transform infrared spectroscopy (ATR-FTIR) spectra; and **(d)** TG curves of the ZIF-8, PSf-30 membranes, 10 wt% ZIF-8/PSf-30, and PDA-2/10 wt% ZIF-8/PSf-30 MMM.

The PDA modified method can overcome the limitations caused by the traditional self-assembly, entrapment, and chemical binding methods (Lu et al., 2017; Wang et al., 2019). After the PDA modification, the stability of MMMs could be improved, and the defects of surfaces will be repaired (Liu et al., 2013; Huang et al., 2014; Yuan et al., 2014). In order to further study the gas separation of MMMs after PDA coating with different time, the H_2_ and CO_2_ gases were selected as the representatives. Figure 5A showed the H_2_ and CO_2_ permeabilities and H_2_/CO_2_ selectivity of 10 wt% ZIF-8/PSf-30 MMM with the different PDA coating time. Either H_2_ or CO_2_ permeability follows a decreasing trend when increasing PDA coating times. This is because the improved denser layers of MMMs and the enhanced gas transport resistance. However, for smaller kinetic diameter gas such as H_2_, the permeability inhibition was not obvious than that of CO_2_. Hence, the H_2_/CO_2_ selectivity of MMMs was improved, such as the selectivity of PDA-2/10 wt% ZIF-8/PSf-30 MMM achieved 9.3 at 4 bar and 30°C. Figure 5B showed the H_2_ and CO_2_ permeabilities and H_2_/CO_2_ selectivity of PDA-2/10 wt% ZIF-8/PSf-30 MMM as a function of feed pressure. H_2_ and CO_2_ permeabilities of PDA-2/10 wt% ZIF-8/PSf-30 MMM change slightly with increasing feed pressure. However, the H_2_/CO_2_ selectivity decreased significantly when the pressure increases, which is due to the CO_2_ permeance improvement. This can be attributed to enhanced CO_2_ solution into the PDA coating layer at high pressures (Huang et al., 2014; Wang et al., 2015).

**Figure 6:**
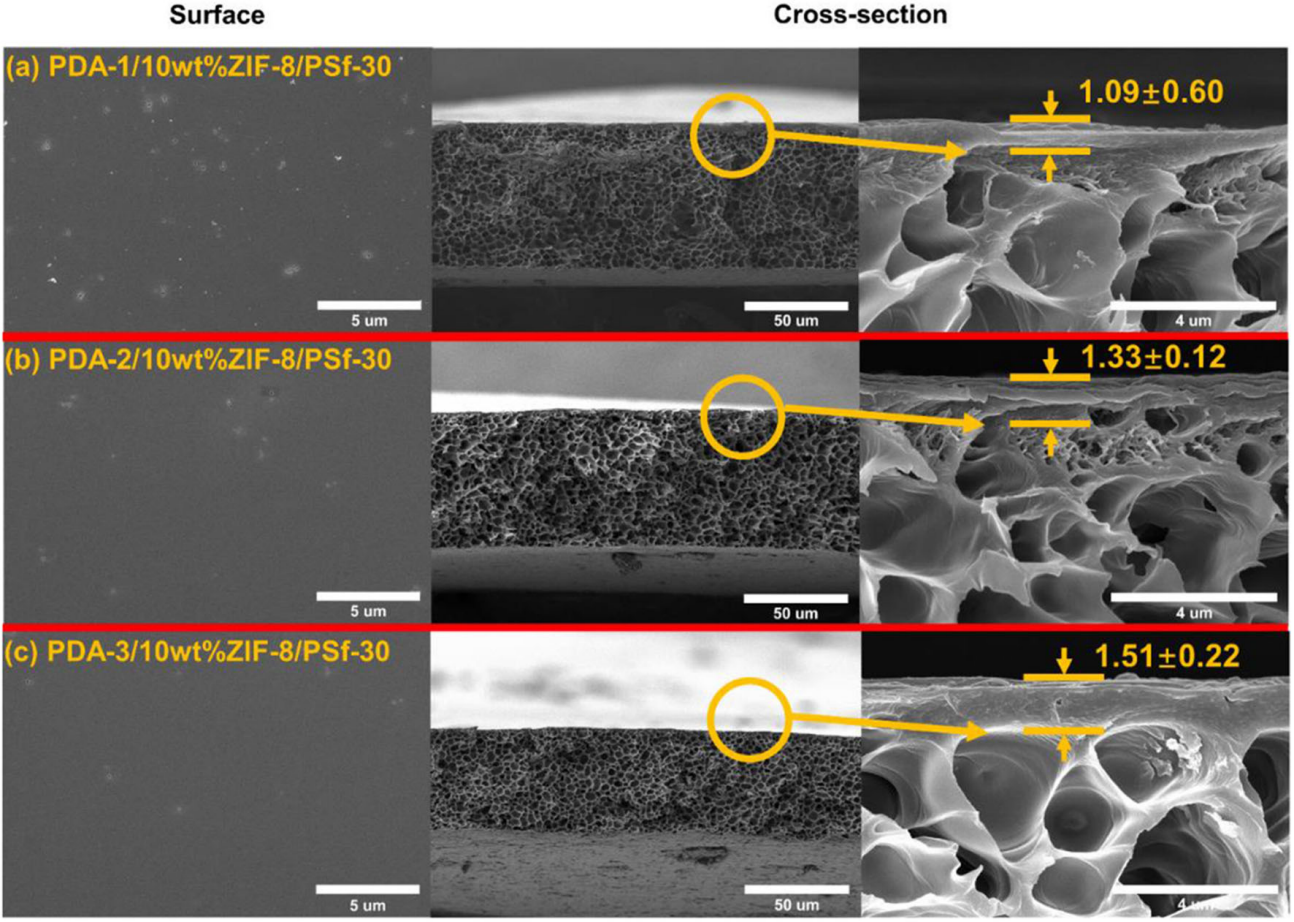
Surface and cross-section of the 10 wt% ZIF-8/PSf-30 MMMs with different PDA coating times. **(a)** 1 h; **(b)** 2 h; and **(c)** 3 h.

In addition, the surface property of PDA-2/10 wt% ZIF-8/PSf-30 MMM was investigated by ATR-FTIR as shown in Figure 5C. Compared with the 10-wt% ZIF-8/PSf-30 MMM, a different peak at 1,540 cm^−1^ was attributed to the N–H bending vibration of PDA (Habibi et al., 2015; Zhou et al., 2015), which proved the successful PDA modified layers. However, the TG curves showed good thermal stability of MMMs as shown in Figure 5D. The surface and cross-section SEM images of the 10-wt% ZIF-8/PSf-30 with different coating times are shown in Figure 6. The SEM images were taken to investigate the changes in surface morphology of MMMs with different coating times. With increasing PDA coating time, it can be observed that the defects in the surface almost disappeared, and the little rough surface becomes quite smooth.

**Figure 7:**
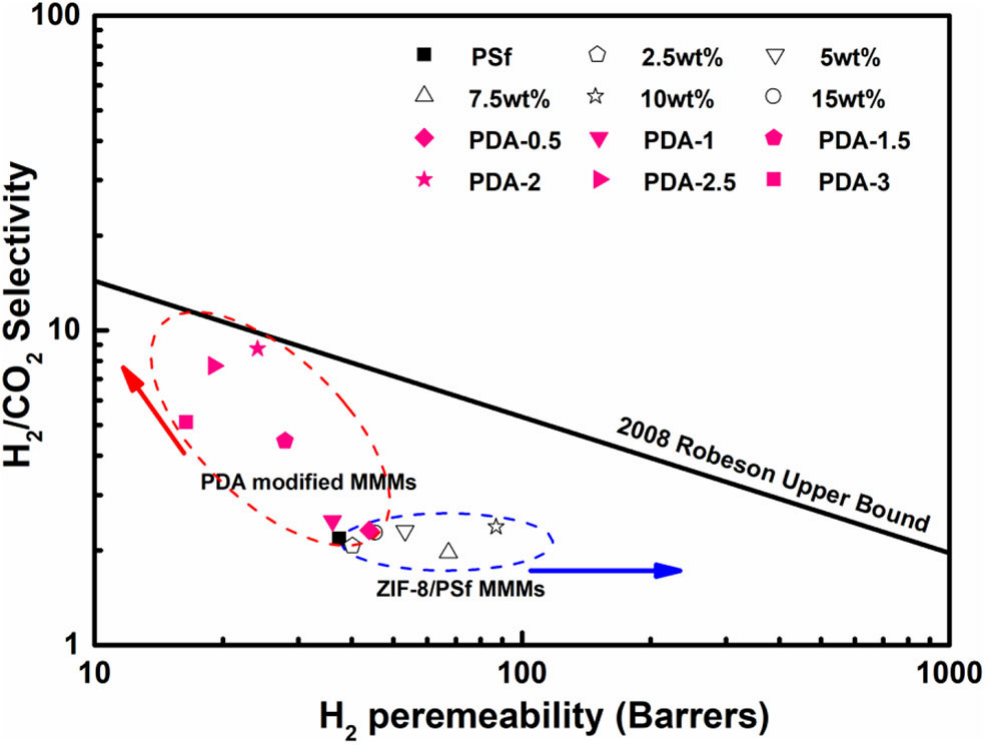
Selectivity of H_2_/CO_2_ vs. H_2_ permeability for the prepared MMMs at 30°C under four bar in the presence of 2008 Robeson upper bound.

The correlation between selectivity and permeability for H_2_/CO_2_ is shown in Figure 7. Embedding ZIF-8 nanoparticles into the PSf-30 asymmetric membrane had a positive effect on the H_2_ permeability. However, the selectivity of H_2_/CO_2_ was not improved. After the PDA coating, the H_2_/CO_2_ selectivity was significantly improved, while the H_2_ permeability was reduced, and the result of PDA-2/10 wt% ZIF-8/PSf-30 MMMs was very close to the 2008 Roberson upper bound. Compared to the reported MMMs, the PDA-modified MMMs showed higher selectivity of H_2_/CO_2_ as shown in Table 3.

**Table 3:** A summary of the H_2_/CO_2_ separation performance of the reported MMMs.

**Polymers**	**Fillers**	**Pressure (bar)**	**Temperature (^**°**^C)**	***P_***H*2**_* (Barrer)**	***α*_*H*2/*CO*2_**	**References**
Polyamide	ZIF-8	3	35	~11.3	4.4	Sánchez Laínez et al., 2018b
PBI	ZIF-8	3.5	35	28.5	13	Yang et al., 2012
PBI	ZIF-8	3.5	35	39	6.8	Yang and Chung, 2013
PSf	ZIF-8	1	35	~55	~2	Sorribas et al., 2014
Matrimid®	ZIF-8	-	-	~35	7	Ordoñez et al., 2010
PSf	SiO_2_	3	35	14	2.6	Pakizeh et al., 2013
PSf with PDA modification	ZIF-8	4	30	23.3	9.3	This work

## Conclusions

The novel MMMs have been developed for gas separation application by the PDA post-modified strategy. The ZIF-8 nanoparticles have been embedded in the PSf asymmetric membranes as the MMMs, and the gas permeability of MMMs was significantly improved. The optimal ZIF-8 concentration of 10 wt% produced an H_2_ permeability of 87 Barrer, but the H_2_/CO_2_ selectivity was only 2.38. The PDA modification has been considered as an effective method for improving the properties of membranes. This binding method can overcome the limitations caused by the traditional self-assembly, entrapment, and chemical binding methods (Lu et al., 2017, 2019). Coating the MMMs with PDA repaired most defects of the surfaces, which reduced the H_2_ permeability of MMMs and improved the H_2_/CO_2_ selectivity. For PDA-2/10 wt% ZIF-8/PSf-30 MMM, the H_2_ permeability was 23.3 Barrer and the H_2_/CO_2_ selectivity achieved 9.3 at 30°C under 4 bar. Prepared PDA-modified MMMs were highly promising for H_2_/CO_2_ separation, owing to the simple manufacturing process and effective improvement. These results demonstrated the availability of the PDA post-modified MMMs for gas separation application.

## Data Availability Statement

All datasets presented in this study are included in the article/ supplementary material.

## Author Contributions

XM designed, analyzed data, and wrote the main manuscript. SY performed the experiments and performance evaluation. PL conceptualized, designed, and edited the main manuscript. YZ and JZ helped design complement experiments and reviewed and edited the manuscript. All authors contributed to the review and approval of the manuscript.

## Conflict of Interest

The authors declare that the research was conducted in the absence of any commercial or financial relationships that could be construed as a potential conflict of interest.
